# Reducing the stigma of mental illness

**DOI:** 10.1017/gmh.2016.11

**Published:** 2016-05-10

**Authors:** H. Stuart

**Affiliations:** Centre for Health Services and Policy Research, Queen's University, Kingston, Ontario, Canada

**Keywords:** Mental illness, stigma, stigma reduction

## Abstract

This paper presents a narrative review of anti-stigma programming using examples from different countries to understand and describe current best practices in the field. Results highlight the importance of targeting the behavioural outcomes of the stigmatization process (discrimination and social inequity), which is consistent with rights-based or social justice models that emphasize social and economic equity for people with disabilities (such as equitable access to services, education, work, etc.). They also call into question large public education approaches in favour of more targeted contact-based interventions. Finally, to add to the research base on best practices, anti-stigma programs are encouraged to create alliances with university researchers in order to critically evaluate their activities and build better, evidence informed practices.

## Introduction

The public health importance of mental disorders has been highlighted by the Global Burden of Disease study, which catapulted mental health promotion and prevention onto the global public health stage. In 1990, five of the top ten leading causes of disability worldwide were from mental illnesses, accounting for almost a quarter of the total years lived with a disability (Murray & Lopez, 1996). More recent estimates indicate that the disability associated with mental and substance abuse disorders has grown from 5.4% of all disability-adjusted years of life worldwide in 1990, to 7.4% in 2010 (Whiteford *et al*. 2014). Estimates from community-based epidemiologic surveys place the lifetime prevalence of mental disorders to be as high as 50% and the 1-year prevalence to be as high as 30%, depending on the country (Kohn *et al.* 2004).

Despite growing recognition of the burden associated with mental illnesses, and the availability of cost-effective treatments, they are not yet afforded the same policy or program priority as comparably disabling physical conditions. The most recent World Health Organization Mental Health Atlas clearly demonstrates the inadequacies of mental health treatment infrastructure worldwide. For example, the average per capita spending on mental healthcare is less than 2 US$ and less than 25 cents in low-income countries. Almost half of the world's population lives in a country with less than one psychiatrist per 200 000 residents. Despite decades of deinstitutionalization, still 63% of the world's psychiatric beds remain in large mental hospitals, known for anti-therapeutic environments and human rights violations, taking up 67% of total spending (World Health Organization, 2011). Data from the World Health Organization's Mental Health Consortium Surveys show that, in developed countries, 35–50% of people with serious mental illnesses living in the community had not received treatment in the year prior to the survey. In developing countries, unmet need was as high as 85% (The WHO Mental Health Survey Consortium, 2004).

Statistics such as these highlight the substantial gap between the public health burden caused by mental illnesses and the resources needed to prevent and treat them, particularly in low- and middle-income countries. In some lower income countries, for example, families cope with the lack of treatment resources by chaining a mentally ill relative to an immovable structure, such as a tree or a bench, where they are open to public scrutiny, teasing, and humiliation. In others, they are caged, beaten, maltreated, or thrown out of their communities where they are mauled or eaten by wild animals (Lee, 2002). As this literature shows, in addition to the symptoms of the illness, people with mental disorders must also endure important structural inequities that impinge on their health, welfare, civic participation, access to resources, and quality of life, and this is particularly true of people with mental illnesses living in middle- or low-income countries where flagrant human rights abuses are common (Arboleda-Florez & Stuart, 2012).

Negative societal responses to people with mental illnesses may be the single greatest barrier to the development of mental health programs worldwide. These pernicious effects, and the associated human rights issues, are increasingly recognized as a worthy target for social action. In recent years, a number of programs have been implemented under the rubric of ‘anti-stigma programming’ to promote greater social equity for people with mental illnesses.

This paper provides a narrative review of anti-stigma programming using examples drawn from different countries to illustrate promising or best practices in the field. The paper does not provide an exhaustive or systematic review of anti-stigma programs, but rather selects programs for the elements that best illustrate the points being made. Large, more recently mounted programs, and those with an evidence base were preferred as they provide current examples of activities in the field. Large national or regional programs are noticeably absent in low- and middle-income countries; however, a number of local implementation projects are discussed to illustrate the potential for transferability of concepts and approaches from high- to low-income settings.

## Stigma defined

Link & Phelan (2001) have noted that considerable variation exists in the scientific literature concerning the definition of stigma. In many instances, it is used vaguely to refer to a mark of shame or disgrace, or to some related concept such as stereotyping or rejection. When it is explicitly defined, Goffman's seminal conceptualization is often used, where stigma is an attribute that is deeply discrediting – one that taints the bearer and reduces their social value. By comparison, Thornicroft (2006) focus on three social psychological aspects of the problem: knowledge, attitudes, and behaviour, while Link and Phelan take a broader, socio-structural view. From this broader perspective, stigma exists when a number of components interact. First, people must distinguish and label a particular human difference (in this case mental illness) as socially salient, resulting in culturally derived categories that are used to differentiate people into groups. Second, labelled differences must be linked to a set of undesirable characteristics thus forming a negative cultural stereotype (or oversimplified characterization) that is summarily applied to every member of the group. Third, those who are so labelled and stereotyped are seen as fundamentally different from the dominant group, creating an ‘us-them’ demarcation. Fourth, stigmatized groups are socially devalued and systematically disadvantaged with respect to access to social and economic goods (such as income, education, housing status), creating poorer health and social outcomes. Discrimination may be experienced in the context of individual interactions, or it may be structural, when accumulated institutional practices create inequities. Finally, stigmatization is entirely contingent on access to social and economic power, as only powerful groups can fully disapprove and marginalize others.

According to this conceptualization, approaches to stigma reduction must be multi-faceted to address the many mechanisms that can lead to disadvantaged outcomes; and multilevel, to address stigma perpetuated at the individual and social-structural levels. Link and Phelan suggest that interventions targeted at only one mechanism (such as employment equity), will be doomed because their effectiveness will be undermined by the broader social factors that are left untouched. They suggest that interventions must either produce fundamental changes in the negative attitudes and beliefs of members of powerful groups, or change the power relations that underlie their ability to act on these attitudes and beliefs (Link & Phelan, 2001). When considering stigma in a global public health perspective, a definition that highlights the serious social and structural forces that create inequities for people with a mental illness is preferred, because this is the stark reality for those living in middle- and low-income countries, where policy, health system, and financial resources systematically exclude people with a mental illness and their family members.

Despite the comprehensive definition offered by Link and Phelan, many anti-stigma programs continue to use the term ‘stigma’ synonymously with attitudes (e.g. Time To Change, http://www.time-to-change.org.uk, see Me Scotland, http://www.seemescotland.org). Advocates such as Everett (2004) or Sayce (2003) have criticized the stigma-as-attitude perspective because it fails to highlight the fact that people with mental illnesses routinely have their civic and human rights violated. They would prefer a rights-based or social justice model that shifts the emphasis away from attitudes to the need for social and economic equity for people with disabilities in all areas of life, including access to health services, education, and work.

## Cross-cultural differences in stigma

Outside of the clear structural inequities in mental health systems and access to care that disproportionately affect low- and middle-income countries (The World Health Organization, 2003), there have been few attempts to directly examine cross-cultural differences in public or personal stigma using common standardized approaches to data collection and measurement. A notable exception is the study by Thornicroft *et al*. (2009) who have documented the personal stigma experiences of 732 people with schizophrenia from 27 developed and developing countries. Fully three quarters (72%) indicated they felt the need to conceal their diagnosis, 64% anticipated they would be discriminated against in applying for work training or education, and 55% anticipated discrimination in close relationships. The effects of discrimination were evident across a broad range of daily experiences such as with family, friends, and employers, across all of the countries studied. In a subsequent analysis, qualitative data from 15 of the participating countries was undertaken (Rose *et al*. 2007). Surprisingly few cross-cultural differences were identified, confirming that personal experiences of stigma are pervasive and a global public health problem.

In 2015, the ASPEN study group (Anti-stigma Program European Network) examined discrimination reported by 1082 people with depression in 34 countries categorized according to their Human Development Index score (Very High; High; Medium/Low) (Lasalvia *et al*. 2015). Participants in high-income countries (with higher human development index scores) were statistically more likely to anticipate being discriminated against, but were no more likely to report having experienced discrimination. Potential reasons for the higher anticipated discrimination in high-income countries include the nature of employment, broader socio-economic context, explanatory models of mental disorders, and self-attribution. For example, almost twice as many individuals living in high-income countries anticipated employment discrimination. In lower income countries, there may be a greater emphasis on family and community ties and higher levels of community support for people who have mental disorders. Explanatory models of mental disorders in lower income countries also place less blame on the individual and the family by attributing causes of mental illnesses to external factors beyond the individual's control such as God's will, Karma, or other supernatural entities. The service user movement in lower income countries is under-developed or non-existent so individuals with mental disorders in these countries may be less aware of the nature of stigma and its consequences. As countries develop, anticipated stigma may increase.

In 2015, Stuart *et al*. (2015) examined the image of psychiatry and psychiatrists among a randomly selected sample of 1057 non-psychiatric clinical teaching faculty across 15 academic teaching centres, the bulk of which were in lower and middle-income countries. A total of 90% of respondents considered that psychiatrists were not good role models for medical students, 84% thought psychiatric patients were unsuitable to be treated outside of specialized facilities, and 73% thought that psychiatric patients were emotionally draining. There were statistically significant differences in stigma scores (calculated as the count of all items endorsed) in only three countries (China, which was lower than average; and Ukraine and Russia, which were higher than average). Country differences explained only 18% of the variation in the mean scale score. These results support the idea that negative attitudes held by professionals are globally pervasive and more similar that dissimilar across countries.

More recently, Seeman *et al*. (2016) conducted a world survey of mental illness stigma using a novel web-based platform that reached more than half a million respondents in 229 countries. This study did not use a standardized stigma measure and probably targeted web-savvy respondents (young, males, with higher education). In the more developed countries (Canada, the USA, and Australia), 7–8% of respondents indicated that people with a mental illness were more violent, compared to 15–16% in developing countries (Algeria, Mexico, Morocco, and China). One can only speculate as to why those in developing countries are more apt to describe someone with a mental illness as violent. As Seeman and colleagues point out, culture, tradition, and access to education and healthcare all shape public perceptions of mental illness. It is difficult to know whether attitudinal or other factors that are associated with the lower treatment gap in high-income countries compared with low, may account for these differences. For example, in developing countries because there is a relative lack of treatment and hospital facilities to prevent or contain potential violence, one might imagine that there is greater exposure to serious mental illness and associated violence in the community. However, in many developing countries, people with more serious disorders are typically managed at home where they may be hidden away to avoid shame and embarrassment, or they may be segregated in large and far away mental hospitals. Those in the community would then represent people with less severe disorders who are less likely to become violent. Despite day-to-day experiences, the public stereotype still may be that the ‘mentally ill’ (defined as those that must be hidden away) are more disturbed and violent. Whatever the explanation, these findings do suggest that the content of public stereotypes may differ depending on country and development level. More research is now needed to uncover the social and cultural conditions that may explain these findings.

There is also evidence that the content of public stereotypes and stigmatizing attitudes differs depending on the disorder group considered. For example, in a random sample of Americans responding to the General Social Survey, vignettes of people with drug or alcohol dependence were more likely to be rated as likely to be a danger to others (over 60% agreed); compared with those who were troubled (<20%), had major depression (30%), or had schizophrenia (60%) (Pescosolido *et al.* 1999). Variation in public attitudes across disorder categories may be even more pronounced in middle- and low-income countries where there is a broader range of explanatory models, including religious-magical views of causation. In a study of 1163 persons in Nigeria, for example, Gureje *et al*. (2006) classified respondents into those subscribing to a biopsychosocial aetiology (84.6%) and those with religious-magical views of causation (15.4%). Knowledge of mental illness was poor in both groups and attitudes were predominantly negative. However, people subscribing to a biopsychosocial view were more likely to believe in the possibility of successful treatment outside of hospital, whereas those with a religious-magical view expressed more tolerant and accepting views. The extent to which specific stereotypic content drives behaviour, resulting in different personal experiences of stigma or structural inequities, is yet unknown.

## The knowledge base for stigma reduction

In most countries, but particularly in middle- and low-income countries, funding for mental health research and evaluation is minimal to non-existent. Consequently, few stigma reduction programs have been subjected to independent review or evaluation. The peer reviewed literature in this area, although growing, remains meager and incommensurate with the hidden burden caused by stigma. Many promising practices have been identified, but few have been implemented widely enough to assess their broad public health effects, their sustainability, their cost-effectiveness, or their transferability from high-income countries, where they largely reside, to low- and middle-income countries, where they may be most needed. In addition, validated fidelity criteria, which identify the active ingredients in a program, are lacking. Identifying the principles and procedures underlying successful anti-stigma programming in such a way that they can be meaningfully tested using rigorous methods and, if found to be successful, widely disseminated, remains an important public mental health priority (Stuart, 2008).

Research is beginning to show that ill-conceived anti-stigma programming can have significant detrimental effects. For example, over the last several decades, a prominent anti-stigma strategy used in high-income countries has promoted neurobiological causes or ‘disease like any other’ messages to the general public. Concomitantly, industry marketing strategies have also provided the public with a wealth information on symptoms, brain-based aetiologies, and specific pharmacological solutions. Rather than reducing stigmatized views, neurobiological explanations have had little or no effect on social intolerance, and in some cases, have deepened it (Pescosolido *et al.* 2010) showing that good intentions are not sufficient to bring about desired change. These findings also suggest that using biological or professional explanations of mental illnesses, as a way of improving knowledge in low- and middle-income countries, where literacy is generally poor, may be ill advised as an anti-stigma strategy.

Many community-based advocacy programs in high-income countries address stigma with good intentions, but with no sound evidence to support their activities (Stuart *et al.* 2014*a*, *b*, *c*). One great challenge in bringing the anti-stigma advocacy community together with the research community is that they have different cultures of knowledge, with different views of what constitutes ‘evidence’. Advocacy groups rarely have the opportunity, the funding, the time, or the expertise to participate in in-depth monitoring, reflection, and learning. They cannot afford to invest in formal evaluation research. Because they need knowledge that is contextualized, easily accessible, decision-oriented, and pragmatic, they accept a much broader range of evidence and share it more informally. Scientific knowledge, which is formal, objective, decontextualized, and peer-reviewed follows a more lengthy process so is of less value in this context (Ferguson, 2005). The challenge is in negotiating how much each side should compromise their view of ‘evidence’ and the evidence gathering process for purposes of program development and evaluation. In low- and middle-income countries, the challenge is not having different knowledge paradigms, but having limited or no capacity to generate research (i.e. having no knowledge paradigm). According to The Academy of Medical Sciences (2008), a quarter of low- and middle-income countries have no mental health researchers at all, and a further quarter of countries have five or fewer researchers in total. When they exist, mental health researchers in low- and middle-income countries are poorly funded, and have little access to resources such as research networks, fellowships, technical support, or well-resourced libraries.

At least three large national anti-stigma programs have built formal ties with university researchers to conduct evaluations of anti-stigma programming. *The Time to Change* program in the UK has partnered with researchers from the Institute of Psychiatry at King's College in London. Working together, they have crafted an extensive evaluation plan and produced evidence-based reviews of the program's activities. Changes in public attitudes were measured every year from 2008 to 2012 using items from the Community Attitudes towards the Mentally Ill Scale, the Opinions About Mental Illness Scale, and two new psychometrically validated scales: the Mental Health Knowledge Schedule and the Reported and Intended Behaviour Scale. In addition, The Discrimination and Stigma Scale was used to assess discrimination experienced by people using mental health services across England. A content analysis of media coverage and an economic analysis, to assess the return on investment, were also conducted (Hederson & Thornicroft, 2013). The results were mixed. There was a small reduction in the discrimination reported by service users, there was no improvement in the knowledge or behaviour of the general public, but there was improved employer recognition of common mental health problems. There were also improvements in medical students' attitudes, though these were short lived, pointing to the need for ongoing programming (Smith, 2013). More detailed results have been published in the scientific literature in a special supplement of the British Journal of Psychiatry (published in 2013).

New Zealand's *Like Minds Like Mine* anti-stigma program has developed strong partnerships with policy makers at the Ministry of Health, an external social marketing firm, as well as researchers from the Institute of Psychiatry in the UK. They assessed the personal experiences of discrimination among mental health service users and their opinions as to whether discrimination had improved over the previous 5 years. Using a modified version of the Discrimination and Stigma Scale developed by the UK-based researchers for *Time to Change*; they surveyed a representative sample of service users selected by officials at the Ministry of Health. Most common discrimination experiences came from family members (30%) and making or keeping friends (28%). A total of 16% of participants identified mental healthcare staff as ‘moderately’ or ‘a lot’ discriminatory, and this was higher (26%) among those who had more than 25 mental health contacts in the previous year. Just over half (54%) had reported that there had been some improvement in stigma and discrimination over the previous 5 years, and 48% considered that the *Like Minds Like Mine* program had assisted in reducing discrimination (Thornicroft *et al*. 2014).

Canada's *Opening Minds* anti-stigma initiative has developed formal partnerships with researchers at five universities across Canada and an extensive network of community providers. Each researcher is responsible for working with research staff (who are funded by *Opening Minds*) and community partners to develop and execute evaluation approaches targeted to a specific group (youth, healthcare providers, journalists, or workers). All programs use some form of contact-based education where people who have experienced a mental health problem deliver an educational intervention centred on personal recovery stories to promote transformational learning. As in the UK, the Canadian program has also created and psychometrically tested several scales to assess changes in attitudes and intended behaviours (e.g. The Opening Minds Youth Opinion Survey; the Opening Minds Scale for Health Care Providers; the Opening Minds Scale for Workplace Attitudes, and the Scale for Supervisor Attitudes). A large media-monitoring project was also undertaken to assess the content and tone of key newspapers. Finally, at the population level, researchers worked with Canada's national statistical reporting agency to develop a measure of the frequency and impact of stigma among people who had received mental health treatment in the year prior to the survey. Overall, results have been positive illustrating that contact-based education has the capacity to reduce prejudicial attitudes and improve social acceptance of people with a mental illness across different target groups and sectors (Stuart *et al.* 2014*a*, *b*, *c*). The next challenge is how to scale these local interventions to achieve national coverage. More detailed results have been published in a special supplement of the *Canadian Journal of Psychiatry* (published in 2014).

Contributions such as these show that university community partnerships are possible and can lead to important insights that contribute to the development of best practices in stigma reduction. They also form the nexus for knowledge exchange between policy makers, providers, and researchers. In future, partnerships and networks such as these should expand to include young researchers from low- and middle-income countries who require training opportunities, networks of practice, and research collaborations. This would broaden our understanding of how programs developed and implemented in high-income countries might translate into the context of low- and middle-income countries, help provide stable funding for the evaluation of intervention projects in low- and middle-income countries, and play an important role in global knowledge exchange. The challenge will be to find funding to promote these global efforts.

## Outcomes of interest

Traditional approaches to stigma reduction have focused on public perceptions of mental illnesses and the mentally ill. Consequently, there is a wealth of survey research in this area describing public knowledge and attitudes. For example, in a review of the literature published between 1990 and 2004, Angermeyer and Deitrich identified 33 national attitude surveys and 29 local or regional surveys, although mostly from Europe (60%). With few exceptions, members of the lay public demonstrated poor mental health literacy, meaning they were unable to recognize symptoms of mental disorders and were unsure as to where to seek help. Negative, stereotypical attitudes were also highly prevalent (Angermeyer & Deitrich, 2006).

Public expressions of behavioural intentions towards people with a mental illness (a proxy measure of discriminatory behaviours) have also been of interest. In a recent meta-analysis of 72 anti-stigma intervention studies targeting public stigma, representing over 38 000 research participants from 14 countries (predominantly Europe and North America), Corrigan *et al*. (2012), found that 32 studies targeted behavioural intentions (typically social distance), and 44 studies targeted attitudes.

Improving the experiences of those who have a mental illness is increasingly viewed as an appropriate benchmark for judging the success of anti-stigma efforts. A number of new measurement instruments have been developed to capture the nature and consequences of personal stigma, so as to target anti-stigma programs to where they are most needed and to measure their effects (Ritsher *et al.* 2003; Raguram *et al.* 2004; Brohan & Thornicroft, 2010; Stuart *et al.* 2014*a*, *b*, *c*). At least three national anti-stigma programs (The UK, Canada, and Germany) have included measures of the experiences of those who have been stigmatized and published these results in the peer-reviewed literature (Gaebel & Baumann, 2003; Corker *et al.* 2013; Stuart *et al.* 2014*a*, *b*, *c*).

A significant limitation of the conventional approaches to stigma-reduction has been the omission of structural outcomes of change. Structural stigma occurs when institutions intentionally or unintentionally create policies, procedures, or practices that disadvantage those with a mental illness, leading to social inequities (Corrigan *et al.* 2004). The United Nations Convention on the Rights of Persons with Disabilities explicitly recognizes that social disadvantage flows from institutional practices, rather than individual impairments. Signatories to the convention agree to remove structural and attitudinal barriers that interfere with individuals' full and effective social participation (United Nations General Assembly, 2006). An example of a national anti-stigma program with clear structural goals is Scotland's *See Me* campaign (http://www.seemescotland.org) which (a) mobilizes people to work together and lead a movement to end mental health stigma and discrimination, (b) works with people to change negative behaviour towards those with mental health problems, and (c) ensures that human rights of people with mental health problems are respected and upheld.

## Common approaches to stigma reduction

Many activities have been grouped under the rubric of stigma reduction (see, for example, Gaebel *et al.* 2005; Beldie *et al.* 2012). The bulk of the literature pertains to programs implemented in high-income countries. The following examples highlight some of the most common approaches taken by programs to address stigma, either directly as a primary outcome, or indirectly as an assumed by-product of other activities.

## Awareness raising

Awareness raising interventions are typically multi-faceted and occur during a specified time in the year when key stakeholders come together to engage in activities designed to increase the public profile of mental health issues. Often an advocacy organization or a network of organizations is involved. For example, the World Health Organization has designated October 10 as World Mental Health Day (http://www.who.int/mental_health/world-mental-health-day/en/) where all stakeholders working in mental health are encouraged to talk about their work, raise awareness of mental health issues globally, and consider what more needs to be done to make mental healthcare a reality for people with mental illnesses worldwide. Advocacy groups in over 100 countries get involved. Some countries have designated a full week of awareness-raising activities where mental health advocates and stakeholders engage in a variety of events designed to promote public education and awareness. While these often generate numerous activities, it is difficult to know whether awareness-raising programs meet their objectives, as they have not been comprehensively evaluated.

Many awareness-raising activities are designed to open a dialogue about mental health on the assumption that bringing it out of the shadows will improve social tolerance. Stigma reduction is a hoped-for side effect. For example, *Active Minds* is an awareness-raising non-profit organization that targets students in universities with chapters across most of the USA, as well as in Canada, and Ecuador (http://www.activeminds.org). The goal is to reduce the stigma surrounding mental health issues by empowering students to speak openly about their mental health problems through student-run mental health awareness, education, and advocacy. They have designated October 5 as the National Day Without Stigma where they encourage students to watch their language, chalk their support (by chalking supportive messages about mental health across campuses), and reach out to someone who may be struggling with a mental health problem. By raising awareness about mental health they hope to create communities of support and promote help seeking. They also have a Stress Less Week, and Eating Disorders Awareness Week and Veterans and Mental Health initiative.

Bell Canada's *Let*'*s Talk* day is an example of a large national program that uses technology and social media to open a public dialogue about mental illnesses. During one day in January, national celebrities, such as Clara Hughes (a six-time Olympic medalist) and others invite Canadians to join the conversation about mental health and the stigma surrounding mental illnesses. Bell uses the day to raise money for mental health research and community initiatives by donating 5 cents for message sent on the Bell network, thus raising 5–6 million dollars each year. In January of 2015 (5 years after the inception of the campaign), #BellLetsTalk was the number one trend on Twitter in Canada and worldwide with a record 4 775 708 tweets of support (http://www.letstalk.bell.ca). Organizations such as *Time to Change* (http://www.time-to-change.org.uk) and *Bring Change to Mind* (http://bringchange2mind.org) also illustrate the importance of social media to disseminate videos, personal stories, and advertisements designed to normalize mental illnesses. These programs capitalize on the momentum that electronic networking can have to raise awareness and fight stigma.

## Literacy programs

Literacy programs try to improve knowledge about mental illnesses, their signs and symptoms, their treatments, and where to go to seek help on the assumption that reduced stigma will be a natural by-product. For example, *beyondblue* (http://www.beyondblue.org.au), a well-established Australian program, aims to reduce the impact of depression and anxiety in the population by: (a) increasing awareness of depression and anxiety, (b) reducing stigma and discrimination, (c) improving help seeking, (d) reducing the impact, disability and mortality, and (e) facilitating learning, collaboration, innovation and research. In this case, stigma reduction is not the primary outcome of interest, but a means to an end. As with awareness programs, an underlying assumption is that improved knowledge and awareness about stigma and discrimination will arm individuals to take appropriate action. For example, with respect to discrimination by the insurance industry in Australia, *beyondblue* undertook extensive research to document the scope and nature of the problem, then provided information on their web page indicating how insurance companies discriminate and what potential solutions could be implemented to resolve this problem. They also provided information on how individuals could get involved by lodging a complaint or an appeal and where to go for support and legal advice. However, it is not clear whether the information provided by *beyondblue* has resulted in increased insurance equity for people with a mental illness.

Population-based literacy programs often use mass media campaigns to transmit health messages to a wide public audience. Few studies have examined the impact of such campaigns on stigma reduction, and those that have, report mixed, limited or no results. Often, campaigns are judged by the amount of penetration (usually measured by recall or visits to a web site), but even this may be meager. For example Corrigan describes a large campaign in eight pilot sites in the U.S. Beginning in November 2004, monthly visits to the web site tripled from 2743 to 7627, but this translated into an audience penetration of only 0.000061% of the population. In addition, 88% of the visitors exited the web site in <1 min (Corrigan *et al.* 2012). Mass media campaigns may not be cost-effective compared with other more direct stigma-reduction approaches, particularly when baseline levels of literacy are high (Stuart *et al.* 2012). For example, the *Defeat Depression Campaign* that was run in the UK between 1991 and 1996 showed that 97% of respondents did not agree with the stigmatizing statement that depressed people are often mad or unstable, and this changed little over the course of the campaign, no doubt as a result of a ceiling effect. When changes were noted, it was not clear that they were a consequence of the campaign messaging, as <5% of the 1995 sample could remember having heard the campaign and this declined to 2% in 1997 (Francis *et al.* 2002).

Two media campaigns undertaken in Canada as part of anti-stigma programming also failed to show change over time. The first was a radio campaign that was undertaken as part of the Canadian pilot program of World Psychiatric Associations' global anti-stigma program to convey the message that schizophrenia was treatable (Stuart, 2003). Over 500 radio messages narrated by a local psychiatrist including a short story by someone with lived experience of schizophrenia were aired at different times during the day for several months. Pre and post-opinion surveys showed that the proportion of respondents who remembered hearing something on the radio rose from 2% at baseline to 27% at post-test, indicating that the radio campaign was successfully connecting with audiences. However, there was no improvement in knowledge, attitudes, or socially distancing behaviours. In both pre-test and post-test samples the majority (60%) could identify a biological determinant of schizophrenia in an open-ended question, 70% endorsed community-based treatment, and 80% agreed that people with schizophrenia require medications. These results show that audience penetration (here measured by awareness) may not be correlated with key outcomes as is often assumed.

The second campaign was undertaken by the Mental Health Commission of Canada's *Opening Minds* anti-stigma initiative (Stuart *et al.* 2014*a*, *b*, *c*). Various media sources were used to transmit messages emphasizing treatment and recovery, including first-person accounts of people who had experienced a mental illness. Major newspapers, television commercials during prime time television, and social networking were used. No appreciable improvements on any of the survey items were noted. For example, about one-third of the sample agreed that people with a mental illness could make a complete recovery – one of the central messages of the campaign. This increased by only 1.1%. Over half of the sample considered that the average Canadian would feel somewhat or very uncomfortable socializing with someone with a mental illness and this did not change. Based on these results, the program reconsidered the role of media messaging as the main intervention strategy and instead opted for a more intensive and targeted approach to stigma reduction.

As a final example, media interventions have been a central piece of England's Time To Change anti-stigma program with the goal of bringing about a 5% positive shift in public attitudes toward mental health problems and 5% reduction in discrimination over a 5-year period (Mehta *et al.* 2009). The initiative was well funded with 18 million pounds from the Big Lottery Fund and Comic Relief. Each year there were two main bursts of social marketing activity including national television, print, radio, cinema, outdoor advertisements, and online advertisements. The effectiveness of the campaign in improving knowledge, attitudes, and behavioural intentions was evaluated between 2009 and 2001 (Evans-Lacko *et al.* 2013). Moderate levels of campaign awareness were achieved, ranging from 39 to 64%, depending on the burst. At the population level there was no significant longitudinal improvement in overall knowledge, attitudes, or intended behaviours (a proxy for discrimination), perhaps because the time frame for the evaluation (2.5 years) was too short. However, campaign awareness was associated with positive change in all three measures suggesting that campaign messages were effective for certain sub-groups of the population. Results from campaign evaluations suggest that public attitudes are slow to change as a result of media campaigns whether or not specific mental illnesses, such as schizophrenia are targeted, or whether mental illnesses in general are addressed.

In addition to population-wide interventions, literacy-based programs also may be targeted to specific groups or settings. *Mental Health First Aid* (http://www.mentalhealthfirstaid.org) was developed in Australia but is now widely available internationally in 24 countries; both developing and developed. It extends the concept of first aid to help individuals know how to respond if someone is having a mental health crisis. The program is standardized, so that it is applied with considerable fidelity to the originators' intent (Kitchener & Jorm, 2006). Trainees learn how to assess the risk of suicide or self-harm, listen non-judgmentally, give reassurance and information, encourage the person to get appropriate professional health, and encourage self-help strategies (Jorm *et al.* 2004).

Kitchener & Jorm (2006) reviewed the results of three studies evaluating the effects of *Mental Health First Aid* – one pre-test/post-test of the first 210 members of the public taking the course, one randomized controlled trial in the work force, and one cluster randomized trial in a large rural area of Australia. They report that the training resulted in statistically significant improvements in knowledge about treatments, improved helping behaviours, greater confidence in providing help to others, and decreased social distance (which is one indicator of stigma). The social distance measure used three items (willingness to move next door to someone with a mental illness; spend an evening socializing with someone with a mental illness; and start working closely on a job with someone with a mental illness) resulting in an agreement scale ranging from 5 to 20 with higher scores reflecting higher social distance. Results showed statistically significant reductions in scale scores for all three vignettes describing someone with a mental illness, suggesting that stigma reduction was a by-product of the course. However, effect sizes for the social distance measures were too small to be practically important. For example, in the pre-test post-test evaluation of the first 210 participants taking the course in Australia (Kitchener & Jorm, 2002) the effect sizes (Cohen's *d*) calculated from the means and standard deviations reported in the article were below 0.2, indicating that the group means from pre-test to post-test differed by less than 0.2 standard deviations. Similarly, in the cluster randomized trial that trained members of the public in a large rural area, Cohen's *d* calculated from the reported means and standard deviations for pre-test and follow-up scores for the treatment group was also small (0.26). Although disappointing from the perspective of stigma reduction, these findings indicate that improved mental health first aid knowledge did not unintentionally deepen stigma, which could have been an unanticipated side effect of providing clinical information about neurobiology, signs, and symptoms. Therefore, while literacy programs are important from the point of view of mental health prevention, it is unlikely that they can be used as a formal stigma reduction strategy. More detailed comparative research such as that proposed by Moll *et al.* (2015) will shed greater light on this issue.

## Protest

Interventions that use protest are designed to suppress stigma through objection or denouncement. They are often focused at the structural level, attempting to change organizational behaviours and practices. They have been used successfully to take offensive products off of shelves, change the marketing strategies for films, and to take offensive content out of television and entertainment media (Corrigan *et al.* 2001).

The *StigmaWatch* program operated since 1999 by SANE Australia is one example (http://www.sane.org) of a protest-based activity. People with a mental illness, their friends and supporters identify stigmatizing images presented in the media and submit a complaint to SANE. The submission is reviewed using the national guidelines for media industry codes of conduct and, if the report is found to be inappropriate, *StigmaWatch* informs the media (or business) about the complaint and encourages an amendment or removal of the item. The tone of the letter is firm but respectful, acknowledging that people rarely mean to offend, acknowledging the media guidelines, and requesting that recipients use more responsible portrayals. The majority of recipients respond positively, are often embarrassed; apologize for any offence caused, and promise to think twice in the future. Only a few journalists have responded in negative and dismissive ways. In 2008, the proportion of *StigmaWatch* reports about the media portrayal of depression was 33%. By 2010, this had dropped to 10%, and has since remained at about 5%, suggesting that the program has been successful in improving media reporting (Hocking, 2013).

## Advocacy

Advocacy activities are aimed at inequities that are created by social structures that intentionally or unintentionally limit the rights of individuals with mental disorders. The World Health Organization defines advocacy as a means of raising awareness about the importance of mental health issues and ensuring that mental health is on government agendas (The World Health Organization, 2003). Advocacy employs numerous techniques including awareness-raising, dissemination of information, education, training, mutual help, counselling, mediating, defending, and denouncing. It is designed to ensure that people with a mental illness enjoy the rights and freedoms offered by legislation, and provides avenues of redress for inequitable policies and procedures (Arboleda-Florez & Stuart, 2012).

In 2001, the World Health Organization undertook a major advocacy program by placing mental health on the agenda of the 54th World Health Assembly. A total of 132 ministers of health participated in four round table sessions. At the close, all agreed that limited health budgets could no longer be obstacles for funding mental health services. In addition, on World Health Day that year, local community groups across the world made a special concerted effort to draw attention to mental health issues and advocate for change. On several continents, psychiatric institutions opened their doors to the public to draw attention to the inadequate conditions and human rights abuses in some institutions. Even Pope John Paul II made a public appeal that everyone should commit themselves to defend the dignity and rights of people with a mental illness. Advocacy materials produced by the World Health Organization and national governments were widely disseminated. In China, for example, over 30 000 posters and leaflets, 10 000 brochures, and 40 000 publicity leaflets were circulated. The Pan American Health Organization (the regional office for WHO in the Americas) produced public service announcements that were aired on major networks such as CNN, and WHO Headquarters in Geneva commissioned several videos to demonstrate the role of family in various countries. There were also targeted events for youth, healthcare providers, and decision-makers (World Health Organization, 2001). The outcomes of these activities in reducing stigma are unknown. Though advocacy efforts may be hampered in middle- and low-income countries owing to the lack of non-governmental organizations, the WHO initiatives show that small community groups can work together to help raise awareness of the importance of mental health.

## Social contact

Allport first developed the idea that greater social contact with members of a stigmatized group could replace faulty perceptions and generalizations, and reduce prejudice and discrimination (Allport, 1954). Based on this theory, positive interpersonal contact has been used widely to reduce the stigmatization experienced by people with a mental illness. Corrigan and colleagues recently completed a meta-analysis of 72 outcome studies that used some form of personal contact to reduce stigmatization of people with a mental illness (Corrigan *et al.* 2012). Contact-based education was superior to other more traditional educational approaches in bringing about change. In the more rigorous studies (those that conducted randomized controlled trials), the effect of traditional didactic education in changing attitudes using Cohen's *d* was 0.21, indicating a weak effect, compared to 0.63 for contact-based education, representing a large effect. Behavioural intentions were more difficult to change, but contact was still superior, with a Cohen's *d* of 0.27 (representing a small effect), compared to 0.10 for education (representing a weak effect).

The Mental Health Commission of Canada's *Opening Minds* anti-stigma initiative has made contact-based education a central feature of its activities. The program has developed networks of community-based anti-stigma programs that deliver contact-based education to various target groups such as youth or health providers (Stuart *et al.* 2014*a*, *b*, *c*). The effectiveness of contact-based education has been clearly demonstrated in this initiative, but programs vary in their level of success from large effects to negligible and even negative effects. Consistent with the literature reported above, behavioural intentions have been more difficult to improve, supporting the idea that improved attitudes may be poor predictors of improved behaviours – results that underscore the need for anti-stigma programs to target behavioural change (Stuart *et al.* 2014*a*, *b*, *c*).

## Stigma reduction in low- and middle-income countries

As previously mentioned, there is a paucity of mental health-related research emanating from low- and middle-income countries (McDaid *et al*. 2008). In 2002, Semrau *et al*. (2015) reviewed relevant peer-reviewed and grey literature on stigma related to mental illness in low- and middle-income countries and found few intervention studies. When they existed, they tended to be small and methodologically diverse, with the result that they did not support broad-based interpretations. For example, many countries used leaflets, webpages, newsletters, or reports to improve mental health awareness and knowledge, though few of these were targeted to specific diagnostic groups. In addition, there were some qualitative reports indicating that training programs could improve knowledge and attitudes among primary care staff in Brazil, and among medical students in China. The only large-scale program that incorporated stigma elements was the EMERALD program.

The Emerald program is designed to improve mental health outcomes in six low- and middle-income countries (Ethiopia, India, Nepal, Nigeria, South Africa, and Uganda) by generating evidence and capacity to enhance health system performance and reduce the treatment gap. It does this by identifying key barriers to effective delivery of mental health services within the health system and offering solutions to improve future mental health delivery. To accomplish this, Emerald uses a mixed methods approach to focus on structural factors that create inequities for people with mental disorders; specifically, adequate, fair, and sustainable resources for mental health; integrated provision of services; and improved service coverage. Emphasis is on service user and carer involvement, stigma reduction, and dissemination of research findings (Semrau *et al*. 2015).

Beldie *et al*. (2012) catalogued anti-stigma activities in 14 midsize European countries. Programs and initiatives included under the anti-stigma rubric ranged from changes in legislation, health promotion activities, literacy, and training programs, to advocacy activities. Most programs were poorly and precariously funded, often with support being more symbolic, and of short duration (such as one special awareness day). Even when programs were of longer duration, this did not reflect sustained activity, but bursts of interventions over the course of time. Seldom did they try to empower people with a mental illness or their family members and were often focused on improving knowledge of mental illness among health personnel. Events targeting entire populations did occur and often involved artistic events such as concerts, art exhibitions, or festivals. Best practices in anti-stigma interventions, such as focusing on specific target groups or using social contact to break down social barriers were rarely employed, and results were not rigorously evaluated.

Several small studies outlining the effects of anti-stigma interventions in low- and middle-income countries using models from high-income countries have been published showing promising results (Chan *et al*. 2009; Bayar *et al*. 2009; Worakul *et al.* 2007; Pejović-Milovancević *et al*. 2009; Fung *et al*. 2011). For example, Chan *et al*. (2009) studied the sequencing of education and video-based social contact in ten classes of grade 9 students in Hong Kong. Results showed that video-based contact combined with education were effective in improving knowledge, stigmatizing attitudes, and social distance, but only if the contact video was presented after (not before) the education. Bayar *et al*. (2009) investigated the efficacy of a web-based stigma educational program for residents or specialists in psychiatry in Turkey. Those receiving the emailed educational information that provided an account of stigma demonstrated less socially distancing attitudes towards people with a mental illness. However, of the 918 residents contacted, the majority (713) refused to participate, perhaps suggesting that web-based interventions are not a preferred method of receiving educational materials.

Low- and middle-income countries face important structural challenges with respect to mental health literacy and awareness-raising. Policy makers in low- and middle-income countries place greater priority on infectious conditions, particularly those that result in high mortality. Organized, well-funded mental health systems and researchers capable of evaluating new and emerging programs are lacking (Soltani *et al*. 2016). Another important challenge for anti-stigma activities in low- and middle-income countries is the generally low mental health literacy levels. Non-governmental organizations focusing on mental health are few. Thus, people with mental illnesses and their family members do not have the mechanisms that support community engagement, empowerment, and advocacy, as in high-income countries Members of the general public and even healthcare providers may not agree that certain mental illnesses exist or that they can be treated. A significant portion of the public may also subscribe to religious explanations of mental illnesses that views causal forces as external to the individual. Thus, an important challenge is to devise approaches that increase awareness of the importance of mental health and the burden caused by mental illness, improve knowledge of mental illnesses and their treatability, and promote explanatory models that support best practice interventions (Gureje *et al*. 2006; McDaid *et al*. 2008).

Despite these important structural limitations, the World Psychiatric Association's Global Program to fight stigma associated with schizophrenia was successful in mounting activities in a number of low- and middle-income countries. The success of the program was in outlining broad principles and strategies, rather than proscribing specific activities. This allowed each Local Action Group to explore the nature and consequences of stigma for local residents, prioritize problems that were of importance to people with a mental illness and family members in their local communities, and select targets for action. It proved much easier to find support for a program that was locally relevant and dynamic to changing needs, than one that was fixed and imported from afar. Working with people who have a mental illness and their families was another key to success. In addition, the most successful programs included members of each target audience. Finally, the more defined the target audience, the more directly the messages could address their needs. In most cases, activities were directed toward people with schizophrenia and their families, but in some locations, a more generic approach was taken. This is a good example of how a program can define broad parameters that can be adapted to local contexts (Sartorius & Schulze, 2005).

## Implications and lessons learned

These examples highlight a number of important implications that can inform better anti-stigma practices. First, though the stigma attached to mental illnesses appears to be universal, it plays out indifferent ways according to local contexts. While the prevalence of stigma may be similar across countries, the experience of someone with schizophrenia in the USA or UK, will not be that of someone from a low-income country where mental health systems are rudimentary or lacking, flagrant human rights abuse may abound, research on best practices is lacking, and local advocacy structures are non-existent. Stigma in both high- and low-income countries seems to be fuelled by misunderstandings of mental illness aetiology, stereotypic beliefs, and lack of political will to appropriately fund integrated mental health systems. However, specific methods of addressing these may differ depending on the cultural context. Programs that can set broad principles and strategies hold the most promise for adapting to local contexts and needs. Programs that hold participants to rigorous fidelity criteria (such as Mental Health First Aid) may be unable to address the needs of those in low- and middle-income countries.

Second, it is important for programs to recognize that improving mental health literacy and stereotypic attitudes will not necessarily lead to greater social tolerance or improved social equity. Targeting the behavioural outcomes of the stigmatization process – both at the individual and the institutional levels – is necessary in order to promote full and effective social participation for individuals with a mental illness. Particularly in middle- and low-income countries, this is hampered by the lack of non-government organizations, poor capacity to conduct research, and lack of mental health system capacity.

Third, large social marketing approaches to improve public attitudes are expensive, have yielded mixed results in high-income countries, and may be entirely inappropriate in middle- and low-income countries with fewer resources and less access to technology. More targeted contact-based interventions have shown greater possibilities of improving attitudes and reducing social distance and there is some limited evidence that contact-based approaches can work in both high- and low-income settings. However, there is still much to learn about identifying the unique socio-cultural factors that contribute to stigma in order to improve the transferability of anti-stigma approaches from high-to-low- and middle-income countries.

Fourth, community–university alliances are important in order to critically reflect on the workings of anti-stigma programs, so that this information can be published, thereby adding to the small but growing evidence base on better or best practices in anti-stigma programming. These alliances also form important bridges between the academic, policy, and practitioner communities, which provide a unique platform for discussion and knowledge exchange. The global alliances established as part of the Open-the-Doors program provides an example of how scientists and world leading experts in the field of stigma reduction can partner with a range of advocates from developing and developed countries (Sartorius & Schulze, 2005).

## Future challenges

We know that the severity of public stigma varies depending on the diagnostic group with the more serious mental illnesses, such as schizophrenia, and substance use disorders having higher stigma (Pescosolido *et al.* 1999). We have seen the importance of targeting anti-stigma programs to particular population groupings (such as youth or healthcare providers), but it is not clear to what extent anti-stigma programs also should be targeted to specific disorder categories. The World Psychiatric Association's Global Program to Fight the Stigma of Schizophrenia deliberately chose a targeted approach on the assumption that lessons learned would be transferrable to less stigmatized disorders (Sartorius & Schulze, 2005). Similarly Australia's beyondblue targets their activities to individuals living with depression or anxiety (http://www.beyondblue.org.au). A strength of the focused approach is that it makes it easier to design targeted programs, particularly if there is a knowledge based component that is disorder specific, as well as partner with existing non-governmental organizations and community groups that tend to focus on specific disorder groups (Sartorius & Schulze, 2005).

Little is known about best practices in anti-stigma programming that would apply to low- and middle-income countries, where the bulk of people with mental disabilities live. It is not clear whether approaches used in high-income countries will translate well into settings where mental health resources and infrastructures are lacking, mental health literacy is lower, comorbidities with other stigmatized conditions (such as HIV) are higher, and there may be less use of social media. However, the World Health Organization and the World Psychiatric Association have successfully implemented awareness and anti-stigma programs that have spanned high, middle, and low-income settings. Important to the success of these initiatives has been setting broad principles, building on the activities of local community groups and volunteers, ensuring that activities address problems that are locally important, and allowing flexibility in the way programs are implemented.

Future research examining the nature of stigma across cultural settings is needed in order to understand how unique social factors may influence the nature of stigma and the feasibility and success of anti-stigma interventions (Mascayano *et al*. 2015). Multi-country stigma networks, such as the Indigo project (Thornicroft *et al*. 2009) that examined personal experiences of discrimination by service users with schizophrenia in 27 low-, middle-, and high-income countries hold considerable promise. Because knowledge exchange is a two-way street, it is important to remember that research from middle- and low-income countries will help high-income countries provide more culturally appropriate programs in their increasingly multi-cultural settings. Decreasing mental illness-related stigma and the hidden burden of mental illness worldwide will take a concerted global effort.
